# Prevalence of H. Pylori in Patients with Nasal Polyposis in Vali Asr Hospital, Southern Iran

**Published:** 2011-06-01

**Authors:** F Khajeh, M H Motazedain, Z Safarpoor, M H Meshkibaf, B Miladpoor

**Affiliations:** 1 Department of Pathology, Fasa University of Medical Sciences, Fasa, Iran; 2 Department of ENT, Fasa University of Medical Sciences, Fasa, Iran; 3 Medical Student, Fasa University of Medical Sciences, Fasa, Iran; 4 Department of Biochemistry,Medical School, Fasa University of Medical Sciences, Fasa, Iran

**Keywords:** Prevalence, H.Pylori, Nasal polyposis, Iran

Dear Editor,

Nasal polyp is a common chronic inflammatory disease of the sinonasal mucosa that is histologically characterized by infiltration of eosinophils and large quantities of extracellular edema. It causes considerable morbidity such as nasal obstruction, rhinorrhea and sleep disorders. Pathogenesis of nasal polyps is still debatable.[1][2] Helicobacter pylori may play a role in the pathogenesis of nasal polyposis, chronic otitis media and chronic rhino-sinusitis.[3]The aim of this study was to investigate any correlation between H.pylori infection and nasal polyposis using serological tests and the rapid urease test to determine the seroprevalence of IgG antibody and detect urease enzyme of H. pylori in these patients.

From September 2008 to December 2009, twenty six patients (18 males and 8 females) who referred to Vali-Asr Hospital in Fasa, Southern Iran with nasal polyposis were considered as case group and 20 patients (6 males and 14 females) who referred for nasal septoplasty were regarded as control group.The control group did not have any nasal polyposis, chronic otitis media, allergy, dyspepsia and peptic ulcer based on history and physical examination. The diagnosis of nasal polyp was made by clinical examination,computed tomography and was confirmed by pathological examination. Patients with the history of peptic ulcer and those who were on H2 blockers, proton pump inhibitor or antacids and also every patient with any chronic disease such as asthma and COPD were excluded. Five ml of the venous blood sample of each person was collected, and then serum was separated and stored in -20ºC until the test time.

All samples were analyzed for detection of serum immunoglobulin G (IgG) against H. pylori with Enzyme Linked Immuno-Sorbent Assay (ELISA), (MONOBIND kit, made in USA), in Hamzeh Lab, Fasa, Southern Iran. We used the rapid urease test on a small piece of fresh nasal polyp measured 0.2 in 0.2 cm in case group and the same size piece of nasal mucosa in the control group. The results were shown in Table 1.

A significant relationship was noticed between nasal polyposis disease and H. pylori infection (p=0.001).The rapid urease test showed 19 and 17 positive results in case and control groups respectively that were not statistically significant. The etiology of nasal polyposis has not still been defined; recently the importance of the H. pylori has increased its rating as a considerable issue. Recently colonization of H. pylori in sinonasal mucosa has reported in some articles. H. pylori can induce hypoxia and acidic environment in sinonasal mucosa which facilitate more growth of this microorganism.H. pylori may play an antigenic role which evoked infiltration of inflammatory cells and release of chemical and inflammatory mediators.[4][5][6] As overall,there are three hypotheses for presence of H. pylori in sinonasal mucosa:

1. Sinonasal mucosa is the primary source for H.pylori.

2. Oro-nasal reflux transits H. pylori to the nose and middle ear.

3.Gastro-esophageal acidic reflux transport H.pylori to sinonasal area.[6]

Ozcan C et al. showed any significant relationship between H.pylori and nasal polyps by Immuno histochemistry and Elisa method.[7] Koc C et al. reported that H.pylori was detected in six nasal polyp specimens out of 30 patients using Immuno histochemistry test which was similar to Cengiz et al conclusion. They reported that, H. pylori was found with increased prevalence in nasal polyps. Dines et al. suggested that H.pylori has more a reservoir function than a pathogenic agent.[8] Mornika S et al. found H.pylori in sinus mucosa of some patients with chronic sinusitis by urease test.[9] Kaviani et al.reported 27 (66.2 %) versus 2(10 %) positive result by Elisa test for H. pylori antibody checking and rapid urease test in case and control group respectively, and they suggested significant relationship between H.pylori infection and nasal polyposis.[10] The results of our study that is in agreement with Kaviani and coworkers report, with significant higher seroprevalance of H. pylori in case 15 (57.7 %) versus 2 (20 %) in control group, analyzed statistically by Chi-Square test. This study let us to conclude that, although we found higher seroprevalence of H. pylori is in patients with nasal polyp, anyway more advanced studies are needed to detect the potential role of H. pylori in development of nasal polyposis.

**Table 1:** Positive and negative H.pylori IgG in patient with nasal polyposis and control group

**Group**	**Positive (%)**	**Negative (%)**	**Total (%)**
**case**	15 (57.7)	11 (42.3)	26(100)
**control**	2(10)	18 (90)	20(100)
**total**	17(36.9)	29 (63.1)	46(100)
